# Wavelet-based study of valence–arousal model of emotions on EEG signals with LabVIEW

**DOI:** 10.1007/s40708-016-0031-9

**Published:** 2016-01-21

**Authors:** Seda Guzel Aydin, Turgay Kaya, Hasan Guler

**Affiliations:** Department of Electrical-Electronics Engineering, Faculty of Engineering, University of Firat, Elazig, Turkey

**Keywords:** EEG, Emotions, LabVIEW

## Abstract

This paper illustrates the wavelet-based feature extraction for emotion assessment using electroencephalogram (EEG) signal through graphical coding design. Two-dimensional (valence–arousal) emotion model was studied. Different emotions (happy, joy, melancholy, and disgust) were studied for assessment. These emotions were stimulated by video clips. EEG signals obtained from four subjects were decomposed into five frequency bands (gamma, beta, alpha, theta, and delta) using “db5” wavelet function. Relative features were calculated to obtain further information. Impact of the emotions according to valence value was observed to be optimal on power spectral density of gamma band. The main objective of this work is not only to investigate the influence of the emotions on different frequency bands but also to overcome the difficulties in the text-based program. This work offers an alternative approach for emotion evaluation through EEG processing. There are a number of methods for emotion recognition such as wavelet transform-based, Fourier transform-based, and Hilbert–Huang transform-based methods. However, the majority of these methods have been applied with the text-based programming languages. In this study, we proposed and implemented an experimental feature extraction with graphics-based language, which provides great convenience in bioelectrical signal processing.

## Introduction

Emotions are one of the most complex and most important features that people have. They constantly guide people in everyday life. They are acting on the decisions that people take. They provide information about human experience. Since emotions are an inseparable part of the people, automatic distinction between them is important. Currently, the most widely used techniques for the discrimination between emotions include facial expression, skin conductance, and brain activity. Recently, studies have focused more on electroencephalogram (EEG)-based emotion recognition [1–9]. Brain produces a low-amplitude electrical signal resulting from the ionic activities that take place in it. Special electrodes are attached to scalp to pick up the electrical signals resulting from ionic current between the neurons. These records are called EEG. EEG measures voltage fluctuations. It has different amplitudes and frequencies. It is not periodic but has some rhythmic frequency. It has a low amplitude that ranges between 1 and 400 µV and wide frequency band which ranges between 0.5 and 100 Hz. EEG is most often used for distinguishing emotions and diagnosing epilepsy, sleep disorders, coma, brain death, etc. [2–4]. EEG is divided into five frequency bands that are delta (between 0.4 and 4 Hz), theta (between 4 and 7 Hz), alpha (between 8 and 13 Hz), beta (between 14 and 40 Hz), and gamma (over 40 Hz). These frequencies emerge while brain is performing different functions.

EEG has been investigated for a long time to distinguish different emotions. Many researchers have intended to analyze and extract features of EEG to classify emotions. They use different stimuli paths to evoke emotions such as auditory and visual components, or audio-visual [5–13]. Murugappan et al. studied human emotion (disgust, fear, happy, surprise, and neutral) recognition in EEG with discrete wavelet transform via db4 wavelet function [7]. Features were obtained by the conventional method and energy-based features. Liu et al. proposed real-time fractal dimension based on algorithm of quantification of basic emotions using valence–arousal emotion model [8]. Fear, frustration, sad, happy, pleasant, and satisfied emotions are stimulated by music and sound. Lokannavar et al. introduced emotion recognition system for emotions such as happy, relax, sad, and fear, based on EEG signal through auto regression (AR) and Fast Fourier Transform (FFT) [9]. Lin et al. investigate whether there is a link between four emotional states (joy, happy, sadness, and pleasure) and EEG activity during listening to music [6]. They made a correlation between emotion and EEG that is derived from electrodes near frontal and parietal lob. Polat et al. investigate the reflection of emotions based on different stories onto EEG [10]. The study contains fifteen different emotions that were stimulated by fifteen different stories. They observed distinction in different emotions between 5 and 8 Hz. Kvaale proposed an artificial neural network model to detect emotions [11]. Two-dimensional model of emotion was used. Schmidt et al. investigate whether the pattern of EEG activity distinguished emotions induced by musical excerpts [12]. Musicals were selected according to valence and intensity value. They found that the valence value of musical excerpts was distinguished in asymmetrical frontal EEG activity. The frontal EEG activity might separate emotions in valence and intensity values according to their study.

The aim of this paper is to investigate LabVIEW-based feature extraction methods to make a correlation between EEG and emotions. The structure of this paper is as follows: Sect. 2 includes a brief description of emotions, database signals taken from LabVIEW, and wavelet analysis. Third section includes evaluation of results. The last section involves conclusion and future works.

## Materials and methods

### Emotions

Emotions have been caused by internal and environmental influences. They are complex psychophysiological changes and involve so many different factors. Defining emotions is one of the difficult concepts. It is very difficult to distinguish emotions from each other. Because they are expressed differently in every culture and language, there is no clear distinction between them. There are different opinions about the number of emotions. Since the number of emotions increases or decreases for languages, races, religions, and cultures, it is hard to find the exact number of emotions. However, there are some emotions all people have regardless of cultures and languages such as joy, fear, angry, and sadness. Therefore, while analyzing emotions and classifying them, commonsense emotion should be selected or common model should be used. Today, many researchers prefer to use two-dimensional model owing to less complexity. There are two fixed perpendicular directed lines in this model. Horizontal axis shows valence and vertical axis shows arousal. Emotions are specified by their position in this model. High arousal refers to the excitement and low arousal refers to calmness. Valence is used as a measure of satisfaction such that low value represents sadness and high value represents happiness.

### Database

The data required for the study were taken from DEAP dataset that is a dataset for emotion analysis using EEG, physiological, and video signals [14]. This database consists of two parts. In this study, the second part of the database was used. In the second part, 40 videos that can create different emotions in people were watched by 32 volunteers. These videos have been tagged with about 15 different emotions such as happy, fun, sad, sentiment, relaxation, etc. EEG signals were recorded and each participant rated the videos while watching them. The valence–arousal–dominance–liking–familiarity model has been used in the database for rating criteria. Valence is used as a measure of satisfaction. It is rated between 1 and 9 float type numbers. For example, low value represents sadness and high value represent happiness. Arousal is rated between 1 and 9 float numbers, which refer to the excitement and calmness. The dominance expresses the intensity of emotions. It is rated between 1 and 9 (float numbers). The liking is rated between 1 and 9 (float). In liking section, users are asked how much they liked the videos. The familiarity is rated (integer) between 1 and 5. In familiarity section, users are asked how often they watch videos. EEG signals were recorded according to 10/20 system. There are 32 .bdf format files, each of which has 48 recorded channels at 512 Hz. Then the data were down-sampled to 128 Hz. 32 channels of each file contain EEG records. The remaining are peripheral and status channels. LabVIEW can use .bdf file format, and file format converter (FFC) application can convert it into TDMS file type which is compatible with LabVIEW.

**Fig. 1:**
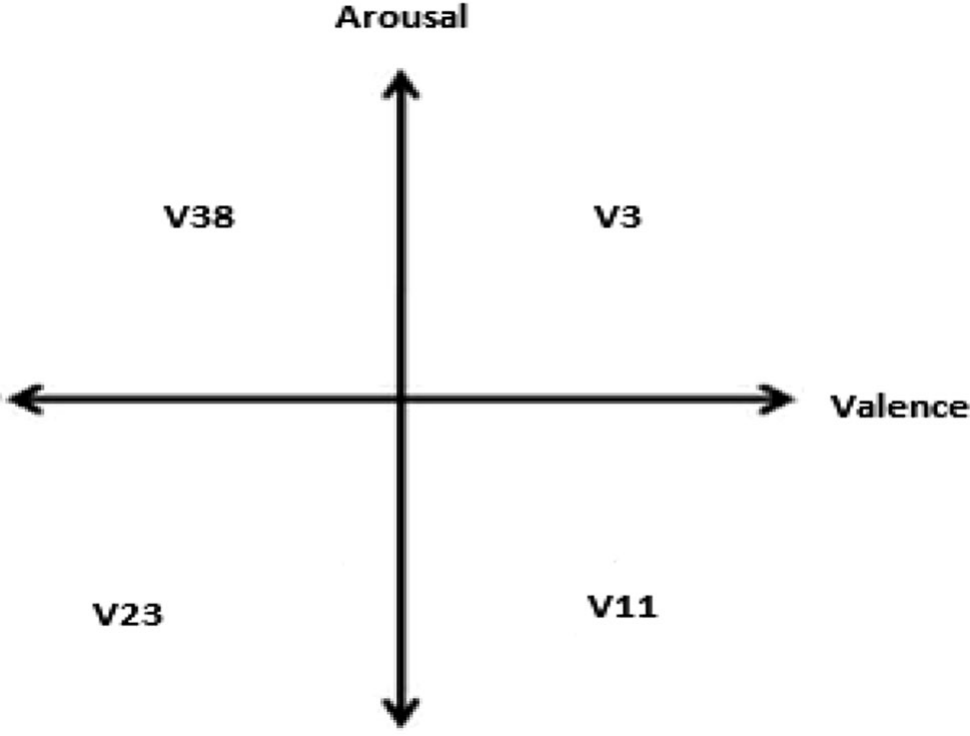
Selected videos at valence–arousal model

In this paper, a two-dimensional model was used. Four videos were selected for this study. The following criteria were identified for selection: high arousal–high valence (HA–HV), low arousal–high valence (LA–HV), high arousal–low valence (HA–LV), and low arousal–low valence (LA–LV) values. The average values for valence and arousal of 40 videos that participants rated were calculated. Four videos were selected according to the calculation result. These are V3 tagged happy for HA–HV, V11 tagged joy for LA–HV, V23 tagged melancholy for LA–LV, and V38 tagged disgust for HA-LV as shown in Fig. 1. Participants 2, 8, 12, and 28 were selected for the study. EEG data were recorded while the participants were watching V3, V11, V23, and V38 videos.

### Improved graphics-based program LabVIEW

LabVIEW is a graphics-based software platform. It has been developed by the American National Instruments Company. The usage of program is increasing in engineering applications and in signal processing applications [15–18]. It provides a visual platform for the development of algorithms. The design and operations of LabVIEW are modeled by physical elements such as oscilloscopes and multi-meter. So it is called a virtual instrument. It is easier to use than the text-based programs. Therefore, the number of users is increasing gradually. Due to graphical representation and Biomedical Toolkits, it is highly preferred especially in biomedical fields.

**Fig. 2:**
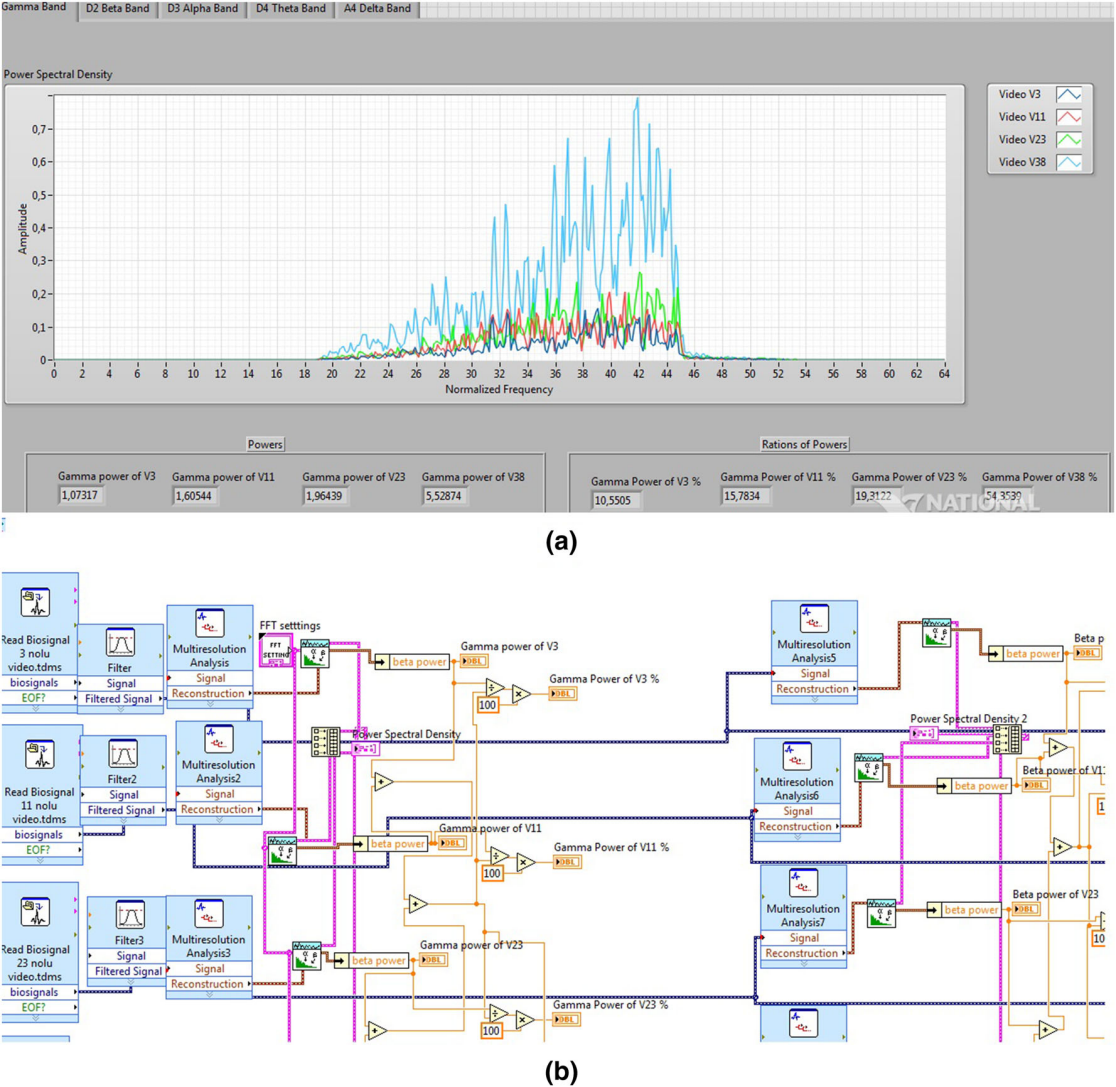
Front panel (**a**) and block diagram (**b**) of LabVIEW

LabVIEW has two parts, the front panel and the block diagram. Block diagram corresponds to code writing part of text-based programming languages. The front panel is part of the program that was taken out. The program that is developed was created with links of graphical tools instead of writing code. Figure 2a shows the front panel and Fig. 2b shows the block diagram of the program.

The developed front panel has five tabs namely delta band, theta band, alpha band, beta band, and gamma band tabs. Tabs show the power spectral density (PSD) of the corresponding tab’s frequency bands. Each tab has four PSDs that are created for four videos by different colors. For instance, gamma band tabs demonstrate PSD of gamma bands for four videos. PSD of gamma band for V3 has been shown with dark blue, V11 with red, V23 with green, and V38 with light blue as shown in Fig. 2a. Figure 2b shows some parts of the graphical code to obtain the features available on the front panel.

Graphical user interface has been created in LabVIEW 15.0 version. FFT, wavelet transform, biomedical toolkit, and advanced signal processing toolkit of LabVIEW platform have been employed to analyze EEG signal.

### Preprocessing

EEG signal has vital information about the operation of the brain and body. However, raw EEG signal is very difficult to interpret correctly. Therefore, this signal needs to be processed with appropriate methods [19–22]. Signal processing applications often consist of three stages that are preprocessing, feature extraction, and classification.

Recorded EEG signal is contaminated by noises and artifacts. These noises must be suppressed in order to get correct required information from the signal and prepare the signal for further processing. Therefore, the data were band-pass filtered between 0.1 and 60 Hz.

### Feature extraction

Feature extraction is the operation of describing a set of features. Feature extraction is so important which can provide the most efficient analysis. The main objective is to obtain reliable data for classification and effective analysis of the signals [23–26]. In this study, wavelet-based feature extraction was applied to the EEG records.

### Wavelet analysis

Fourier transform is the most popular transformation that provides very good results in the analysis of stationary signals whose frequency content is unchanged over time. However, it is not very suitable for analysis of non-stationary signals whose statistic characteristics vary with time. Because Fourier transform can show frequency components that signal includes but it is not report that at what times these frequency components occur. The time and frequency information cannot be obtained at the same time. Therefore, although it is a suitable method for the frequency analysis of time-invariant signal, it is not suitable for the analysis of signals in temporary situation. A wavelet function is an oscillating small wave. It analyzes both shape and window. In this analysis method, window size can be changed. Wide window size can be used to analyze low frequencies and narrow window size can be used to analyze high frequencies. Thus, the optimum time–frequency resolution over the entire signal could be achieved. It makes possible to perform a multi-resolution analysis.

**Fig. 3:**
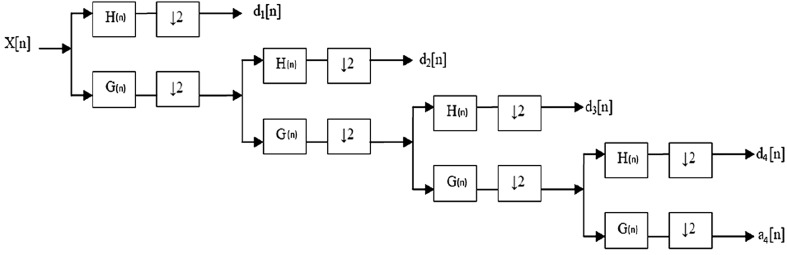
Multi-resolution decomposition of four levels of a signal

**Fig. 4:**
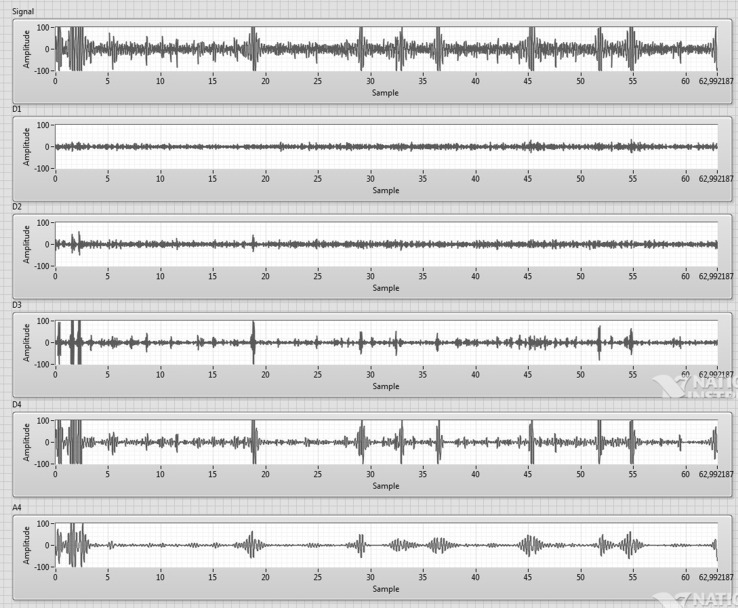
Approximate and detailed coefficients of EEG signal for four-level decomposition

In this study, multi-resolution decomposition of a signal was employed. Multi-resolution analysis allows to view signals at different frequencies with different resolution. The procedure for multi-resolution decomposition of four levels of signals is shown in Fig. 3. Multi-resolution was obtained by filter banks. H(n) represents high pass filter and G(n) represents low pass filter. Thus, the signal is divided into low- and high-frequency components. d_1_[n], d_2_[n], d_3_[n], and d_4_[n] represent detailed coefficients of the x[n] signal and a_4_[n] represents the approximate coefficient of that signal. Figure 4 shows detailed and approximate coefficient of EEG signals. The multi-resolution analysis can be made in arbitrary level depending on the desired resolution. In this study, four-level decomposition is enough to get five frequency bands of EEG signal.

The multi-resolution analysis of dB5 (Daubechies order 5) wavelet function is used for decomposition in this study. EEG signal is divided into four levels and five frequency bands that are gamma, beta, alpha, theta, and delta. This frequency bands are considered to create features. Table 1 shows frequencies corresponding to four levels of decomposition of an EEG signal.

**Table 1:** Frequencies corresponding to four-level decomposition of an EEG signal

Sub-bands	Frequency range (Hz)	Corresponding to EEG frequency band
D1	64–32	Gamma
D2	32–16	Beta
D3	16–8	Alpha
D4	8–4	Theta
A4	4–0	Delta

## Result and discussion

**Fig. 5:**
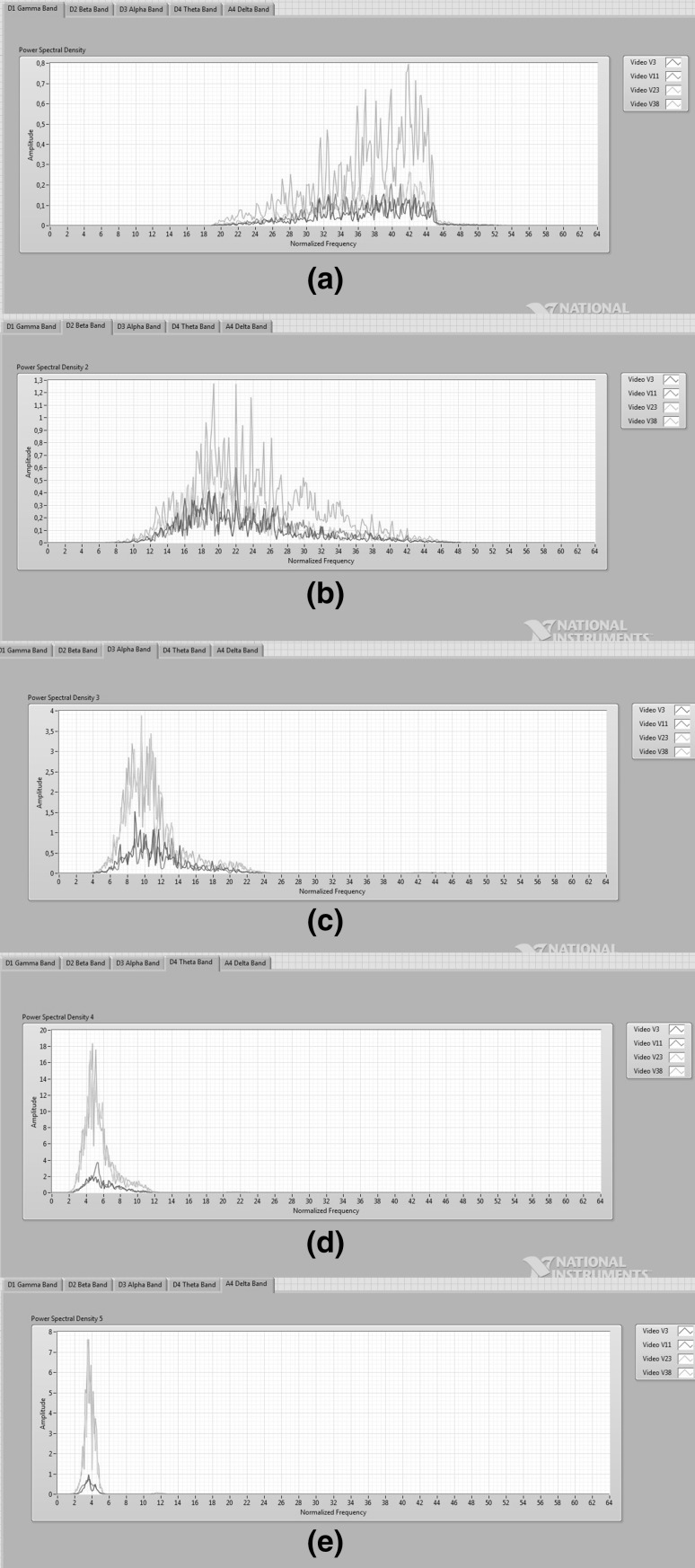
Five frequency bands for four emotions

**Fig. 6:**
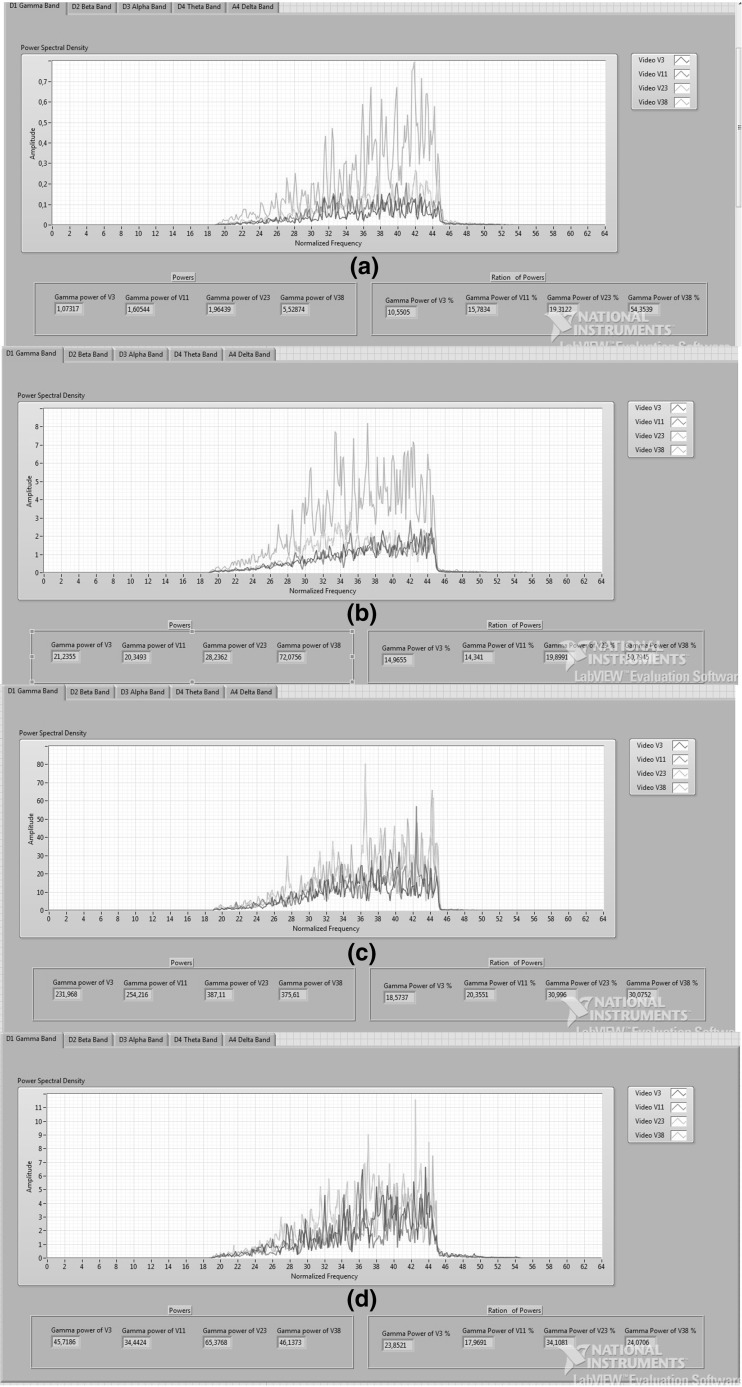
Gamma band of four participants for the videos: **a** PSDs of participant 8, **b** PSDs of participant 2, **c** PSDs of participant 12, and **d** PSDs of participant 28

The wavelet analysis technique was applied to EEG signals to obtain detailed and approximate coefficients. The four-level decomposition of wavelet functions was performed and all the coefficients were used for emotion comparison. Gamma, beta, alpha, theta, and delta frequency bands are considered for deriving features. Figure 5 shows five EEG frequency bands of participant 8 while watching the selected videos. Figure 5a, b, c, d, and e illustrates PSDs of gamma, beta, alpha, theta, and delta bands, respectively, for selected videos. As shown in Fig. 5, PSDs of EEG signals recorded while watching videos labeled happy, joy, melancholy, and disgust have different amplitudes and different average powers of wavelet coefficients. However, the effect of the emotions on different frequency bands cannot be differentiated according to this result. Also making a precise difference is not possible according to the arousal value; however, the effect of the valence value on different frequency bands can be observed. As shown in Fig. 5, the amplitude of PSDs of V3–V11 which have high valence values is lower than that of V23–V38 which have low valence value. The average power of the V23–V38 is greater than that of V3–V11. This analysis was performed for the four participants. The effect of the valence value on EEG can be observed in gamma band for all the participants. Figure 6 shows the PSDs of gamma band of four participants each with four videos. Figure 6a, b, c, and d represents four PSDs of gamma bands that belong to participants 8, 2, 12, and 28, respectively. In the study results, the impact of V23 and V38 videos with a low valence value caused a rise in the amplitude of PSD, while the impact of V3 and V11 videos with a high valence value caused a decrease in the amplitude of PSD of EEG, as shown in Fig. 6. The experimental result indicates that the valence value is most effective to distinguish emotions on a gamma band of EEG. In addition to distinguishing emotions, this study proposes a new reliable working area to analyze EEG signal to extract features. Simple and successful graphical coding can be used for emotion analysis through LabVIEW.

## Conclusion

Emotions play an important role in human life. They teach people how to decide and response. They have a significant role in strengthening relations between people. Therefore, understanding emotions is intriguing for researchers. Despite the fact that a number of studies have been conducted to recognize emotion, majority of them applied text-based programming language.

The object of this study is to investigate the efficacy of different emotions (happy, joy, melancholy, and disgust) on different frequency bands. Different videos labeled happy, joy, melancholy, and disgust were used to stimulate emotions. The data required for this study were taken from DEAP dataset. Emotions used in this study were selected based on the valence–arousal model. Participants 2, 8, 12, and 28 were selected for this study. Db5 wavelet function was applied to divide EEG signal into five frequency bands. When the results are analyzed, the impact of valence value was observed on gamma band. It is briefly reported that emotion effect according to valence score can be achievable with multi-resolution analysis of db5 wavelet-based feature extraction on gamma band. However, there are some limitations on EEG-based emotion recognition. One of the major limitations is that the same emotions do not cause the same effect in each person. So, it is not possible to reach a definitive judgment. In this study, a general conclusion has been reached that emotions having low valence value have the higher amplitude in PSD of gamma band than emotions having high valence values. This study has been conducted through graphical code-based program. Creating program with LabVIEW software is quite easy and simple. It takes very less time to develop a signal processing algorithm, while text-based program takes relatively more time [27–29]. In addition, execution time is shorter than that for text-based programing language in signal processing applications. Moreover, it has many digital signal processing options such as biomedical toolkit and digital signal processing toolkit. The results of this study also show that graphical way of programming has successfully determined emotions according to arousal score.

The future works will include classification methods for further analysis to make specific link between EEG and emotional states. It is expected to understand the details of association between different emotional states and various EEG features. In addition, the effects of different wavelet functions will be investigated for remarkable conclusion.
